# Differences in sarcopenia prevalence between upper-body and lower-body based EWGSOP2 muscle strength criteria: the Tromsø study 2015–2016

**DOI:** 10.1186/s12877-020-01860-w

**Published:** 2020-11-10

**Authors:** Jonas Johansson, Bjørn Heine Strand, Bente Morseth, Laila Arnesdatter Hopstock, Sameline Grimsgaard

**Affiliations:** 1 Department of Community Medicine, UiT The Arctic University of Norway, Postboks 6050 Langnes, 9037 Tromsø, Norway; 2 Department of Chronic Diseases and Ageing, Norwegian Institute of Public Health, Oslo, Norway; 3 School of Sport Sciences, UiT The Arctic University of Norway, Tromsø, Norway

**Keywords:** Sarcopenia, Prevalence, EWGSOP2, Cohort, The Tromsø study

## Abstract

**Background:**

The European Working Group on Sarcopenia in Older People (EWGSOP2) recommends grip strength and chair stand tests to be used as primary defining measures. It is unclear how either test affects prevalence estimates.

**Methods:**

This cross-sectional study involved 3498 community-dwelling participants (40–84 years) from the 7th Tromsø Study survey (2015–2016). We used grip strength, five-repetition chair stands, four-meter Walk Speed Test, Timed-Up-and-Go (TUG) and Dual-Energy X-ray Absorptiometry measurements. Data were analyzed using multiple linear regression models and ROC-curves.

**Results:**

Probable and confirmed sarcopenia prevalence was 1.3 and 4.4% based on grip strength and chair stands, respectively. There was very low agreement between grip strength and chair stand cut-offs (*κ* = 0.07), with only 4.3% of participants defined as having probable sarcopenia overlapping in the two criteria. Participants with grip strength-based sarcopenia had lower mean height, weight, waist circumference, and appendicular lean mass relative to body height (ALM_height_^2^) than non-sarcopenic participants (all *p* < 0.001), after adjusting for multiple covariates. Conversely, participants with chair stand-based sarcopenia had similar height, higher weight, waist circumference and body fat% compared to non-sarcopenic participants (all *p* < 0.05). Area-under-curves (AUCs) for TUG-time were significantly larger when using chair stand instead of grip strength cut-offs (0.86, 95% CI 0.84–0.89 vs. 0.75, 95% CI 0.69–0.83).

**Conclusions:**

Using chair stands instead of grip strength more than doubled probable sarcopenia prevalence across all ages. The two measures defined individuals of contradictory anthropometrics, body composition, and dissimilar physical function to have probable sarcopenia. Researchers should further evaluate the consequences of using different strength measures in the EWGSOP2 definition to classify sarcopenia.

**Supplementary Information:**

The online version contains supplementary material available at 10.1186/s12877-020-01860-w.

## Background

Sarcopenia is a muscle disease characterized by progressive loss of strength, muscle mass and physical function with increasing age [1]. Sarcopenia predicts several adverse outcomes, including falls and fractures, reduced quality of life, cognitive impairment and increased mortality [2–5]. Several studies have shown that regular resistance exercise effectively prevents sarcopenia in older adults, effects that may be further enhanced by simultaneously increasing protein intake [6–8]. However, clinicians rely on adequate disease-defining thresholds and guidelines to direct effective treatments, and implementation of the sarcopenia diagnose is currently hindered by a lack of international consensus over a common operational definition [9].

Since the 1990s, reported sarcopenia prevalence have ranged between 10 to 40% in community-dwelling individuals aged 55 or more [10]. The substantial range of these estimates are likely the result of continuous revisions of the disease definition, parallel establishment of several international sarcopenia work groups and use of different criteria. The revised sarcopenia definition from the European Working Group on Sarcopenia in Older People (EWGSOP2) now recognizes muscle strength as the primary defining measure in contrast to the previous definition that emphasized muscle mass [11, 12]. EWGSOP2 guidelines recommend grip strength or chair stand tests for determination of upper- or lower-body strength respectively, and have called for validation of their new operational criteria in different populations [11]. To ensure proper diagnosis of sarcopenia status, it is important to evaluate how the strength measures perform against each other, as previous research have reported weak associations between strength in upper- or lower-body extremities, in addition to different associations with physical function [13, 14]. It is thus unclear whether grip strength and chair stand tests can be used interchangeably as primary defining measures, and how selecting one or the other may affect sarcopenia prevalence estimates.

We aimed to investigate sarcopenia prevalence according to the EWGSOP2 definition in a Scandinavian population, and to evaluate consequences of using either upper-body or lower-body strength assessment as primary defining measures.

## Methods

### Study design and participants

The Tromsø Study is an ongoing population-based study in Tromsø, Northern Norway, with seven completed waves of data collection since 1974 [15]. The current study analyzed data from the 7th survey (Tromsø 7, 2015–16), with procedures described elsewhere (see also Additional file 1) [16]. We included community-dwelling participants aged 40–84 years with complete data from physical function and body composition measurements (*n* = 3498).

### Strength and physical function assessment

Grip strength testing followed the Southampton protocol procedures [17]. Participants were seated and instructed to hold a Jamar+ Digital Dynamometer (Patterson Medical, Warrenville, IL, USA) in a 90° elbow joint angle, and squeeze the dynamometer with maximal effort. The test was repeated three times per hand, and the present study analyzed the highest of the six values. The dynamometer was brand new and freshly calibrated by the manufacturer.

The five-repetition chair stand test, part of the Short Physical Performance Battery (SPPB) [18], was used for assessment of leg muscle strength. Participants were instructed to completely rise up from a seated position five times as fast as possible, without stopping and to keep their arms crossed over their chest. Participants initially practiced one chair rise before the main test. Using a stopwatch, total time was measured in seconds (s) from the initial seated position until the participant had risen for the last time and were standing up. The test was aborted if participants used their arms for rising, if more than 60 s passed, or if there were uncertainties regarding the patient’s safety.

The Timed-Up-and-Go (TUG) test was used to assess overall physical function, where participants were instructed to rise unaided from a chair (starting point), walk three meters (m) forward, walk back to the chair and be seated again (finish). They were asked to move with a normal, everyday pace and were aided by markings on the floor that indicated where to turn. The total time in s was recorded between the starting point and finish using a stopwatch.

Participants also performed the four-meter walk speed (4MWS) test twice, where they were instructed to walk in their regular pace, stopping by a four-meter marking on the floor. The total time was measured using a stopwatch and we calculated walk speed in m per s (m/s) by dividing the total time by four. The faster of the two trials was used in the analyses.

### Body composition measurement

Body composition was measured by Dual-Energy X-ray Absorptiometry (DXA) using a Lunar Prodigy device (GE Healthcare Lunar, Madison, WI, USA). Participants underwent a whole-body scan lasting approximately 10 min. Trained technicians inspected each completed scan picture and made appropriate adjustments to the regions of interest in accordance with the manufacturer’s guidelines. The device was calibrated each morning using a phantom, and post-scan analyses were performed in enhanced mode using enCore version 17 (GE Healthcare Lunar, Madison, WI, USA). For the present study, we extracted data on appendicular lean mass (ALM; lean mass in arms + legs) and total body fat percentage (TBF%).

### Sarcopenia definition

Probable or confirmed sarcopenia were defined according to EWGSOP2 thresholds [11]. Probable sarcopenia was defined as having grip strength < 16 kg for women and < 27 kg for men, or taking > 15 s to perform five chair stand repetitions for both men and women. Confirmed sarcopenia was defined as further having ALM relative to squared body height (ALM_height_^2^) < 5.5 kg/meters squared (kg/m^2^) for women and < 7 kg/m^2^ for men, in line with EWGSOP2 recommendations to use a standardized approach to this parameter [19]. We chose to analyze the entire sample from age 40–84 in order to investigate and compare how prevalence estimates between grip strength-based and chair stand-based sarcopenia status might progress from middle age to older age.

### Covariates

Weight (kg) and height (m) were measured in light clothing without shoes, and body mass index (BMI) was calculated as kg/m^2^. Waist circumference (centimeter; cm) was measured with a measuring tape at the umbilical level. Participants answered a comprehensive questionnaire including data on level of education (primary, upper secondary, college/university < 4 years, and college/university ≥ 4 years) current smoking and diabetes, and previous cardiovascular disease (CVD; stroke and myocardial infarction). Trained research personnel performed all measurements according to standard procedures.

### Statistical analysis

We reported percentages (%) for sarcopenia prevalence, and used means, standard deviations (M ± SDs) and 95% confidence intervals (95% CIs) to present population characteristics. We standardized total sample prevalence using the European Standard Population 2013 [20]. The Student’s *t*-test was used to compare groups of sarcopenia status for continuous variables and the chi-square test was used for categorical variables. Cohen’s Kappa (*κ*) was used to determine the level of agreement between grip strength and chair stand cut-offs for classification of probable sarcopenia and subsequent confirmed sarcopenia. We used logistic regression to investigate sex differences in sarcopenia components and presented this with age-adjusted *p*-values. We used multiple linear regression models to analyze the independent association between either grip strength or chair stand-based probable sarcopenia, with anthropometrics and body composition variables. In these analyses, we reported unstandardized beta coefficients with 95% CIs and standardized beta (*β*) coefficients. Model 1 was unadjusted while model 2 (fully adjusted) was adjusted for sex, age, smoking status, CVD, diabetes and education. Residuals were inspected for normality ahead of regression analyses. We used receiver operating characteristic (ROC) curves, and reporting of area under curve (AUC) with 95% CIs to inspect associations between sarcopenia parameters and either the grip strength or chair stand criteria. All statistical analyses were performed using Stata software version 15.1 (StataCorp, College Station, TX, USA).

## Results

### Sample characteristics by sarcopenia status

Table 1 shows descriptive data stratified by groups of sarcopenia status: no sarcopenia, probable sarcopenia (defined by meeting the grip strength or the chair stand cut-off) and confirmed sarcopenia (defined by having probable sarcopenia + meeting the ALM_height_^2^ cut-off). As indicated by the 95% CIs, age was linearly increased while height was decreased in the probable sarcopenia group only. Weight, BMI and waist circumference were higher in participants with probable sarcopenia but lower in participants with confirmed sarcopenia, compared to non-sarcopenic participants. Participants with probable sarcopenia had higher TBF% than non-sarcopenic participants, while participants with confirmed sarcopenia had lower. Women with probable sarcopenia had higher ALM_height_^2^ compared to the non-sarcopenic group while women with confirmed sarcopenia had lower ALM_height_^2^. For men, ALM_height_^2^ decreased linearly over probable and confirmed sarcopenia groups respectively. Participants with probable sarcopenia were significantly more likely to be female (75.6 vs. 57.6%), diabetic (15.4 vs. 5.4%) and to have lower education level than non-sarcopenic participants (*p* < 0.001 for all). In addition, participants with probable and confirmed sarcopenia expressed slower walk speed, poorer TUG performance, lower grip strength and poorer chair stand performance compared to participants without sarcopenia.

**Table 1:** Descriptive characteristics of study participants according to sarcopenia status

Variable	All (*n* = 3498)	No sarcopenia (*n* = 3263)	Probable sarcopenia (*n* = 205)	Confirmed sarcopenia (*n* = 30)
Age-standardized prevalence (%)^a^	–	–	4.5	0.7
Age (yrs)	66.0 ± 9.0 (65.7–66.3)	65.6 ± 8.9 (65.2–65.9)	71.6 ± 7.8 (70.5–72.7)	77.5 ± 4.6 (75.8–79.2)
Weight (kg)	77.34 ± 15.0 (76.8–77.8)	77.3 ± 14.9 (76.8–77.8)	79.8 ± 15.8 (77.6–82.0)	62.0 ± 12.8 (57.2–66.8)
Height (cm)	168.6 ± 9.3 (168.3–168.9)	168.9 ± 9.2 (168.6–169.2)	164.5 ± 9.5 (163.2–165.8)	167.7 ± 10.2 (163.9–171.6)
Women (n, %)	2047 (58.5)	1879 (57.6)	155 (75.6)^b^	13 (43.3)
BMI (kg/m^2^)	27.1 ± 4.3 (27.0–27.3)	27.0 ± 4.2 (26.9–27.2)	29.4 ± 5.0 (28.7–30.1)	21.8 ± 2.8 (20.8–22.9)
Waist circumference (cm)^c^	95.0 ± 12.5 (94.5–95.4)	94.7 ± 12.4 (94.3–95.1)	100.6 ± 12.8 (98.8–102.3)	86.8 ± 12.4 (82.1–91.5)
Total body fat percentage (%)	34.7 ± 7.8 (34.4–35.0)	34.4 ± 7.7 (34.1–34.6)	40.5 ± 6.9 (39.5–41.4)	30.5 ± 7.0 (27.8–33.1)
ALM_height_^2^				
Women (kg/m^2^)	6.9 ± 0.9 (6.8–6.9)	6.9 ± 0.9 (6.8–6.9)	7.1 ± 1.1 (7.0–7.3)	5.1 ± 0.3 (4.9–5.3)
Men (kg/m^2^)	8.4 ± 1.0 (8.4–8.5)	8.5 ± 0.9 (8.4–8.5)	8.2 ± 0.8 (7.9–8.4)	6.5 ± 0.5 (6.2–6.7)
Current smoker (n, %)^c^	388 (11.2)	356 (11.0)	29 (14.6)	3 (10.0)
CVD, previous (n, %)^c^	265 (7.8)	239 (7.5)	20 (10.3)	6 (21.3)^b^
Diabetes, current (n, %)^c^	200 (5.9)	169 (5.4)	30 (15.4)^b^	1 (3.5)
Education (n, %)^c^				
Primary/partly secondary	1173 (34.4)	1063 (33.3)	101 (52.9)^b^	9 (31.0)
Upper secondary	937 (27.5)	871 (27.3)	56 (29.3)^b^	10 (34.5)
Tertiary, short	604 (17.7)	578 (18.1)	20 (10.5)^b^	6 (20.7)
Tertiary, long	696 (20.4)	678 (21.3)	14 (7.3)^b^	4 (13.8)
Walk speed (m/sec)	1.2 ± 0.2 (1.1–1.2)	1.2 ± 0.2 (1.2–1.2)	0.9 ± 0.2 (0.9–1.0)	0.8 ± 0.2 (0.8–0.9)
TUG (sec)	8.6 ± 2.3 (8.6–8.7)	8.4 ± 2.0 (8.4–8.5)	11.4 ± 3.2 (11.0–11.8)	12.8 ± 3.4 (11.6–14.1)
Grip strength				
Women (kg)	27.4 ± 5.2 (27.2–27.6)	27.8 ± 4.8 (27.6–28.0)	23.1 ± 6.2 (22.1–24.0)	19.3 ± 7.1 (15.0–23.6)
Men (kg)	46.7 ± 9.0 (46.3–47.2)	47.3 ± 8.6 (46.9–47.8)	36.8 ± 10.5 (33.8–39.7)	29.6 ± 4.0 (27.5–31.7)
Chair rises, 5 repetitions (sec)	9.9 ± 3.4 (9.8–10.0)	9.4 ± 2.3 (9.3–9.5)	17.6 ± 6.7 (16.7–18.5)	16.5 ± 4.1 (15.0–18.0)

Numbers are mean ± SD (95% CI) or n (%). Probable sarcopenia is defined by either grip strength or chair stand cutoffs. Confirmed sarcopenia is defined by ALM_height_^2^ cutoffs. *ALM*_*height*_^*2*^ Appendicular Lean Mass relative to squared body height, *CVD* Cardiovascular Disease (includes myocardial infarction and stroke), *TUG* Timed-Up-and-GO test. ^a^Total sample prevalence standardized according to European Populations 2013. ^b^Significantly different at *P* < 0.01 level from the non-sarcopenic group according to chi^2^ test. ^c^Missing data present: 0.3% for waist circumference, 1.2% for current smoker, 2.7% for previous CVD, 3.4% for current diabetes, 2.6% for education levels. The Tromsø Study 2015–16

### Sample prevalence of probable and confirmed sarcopenia

**Fig. 1:**
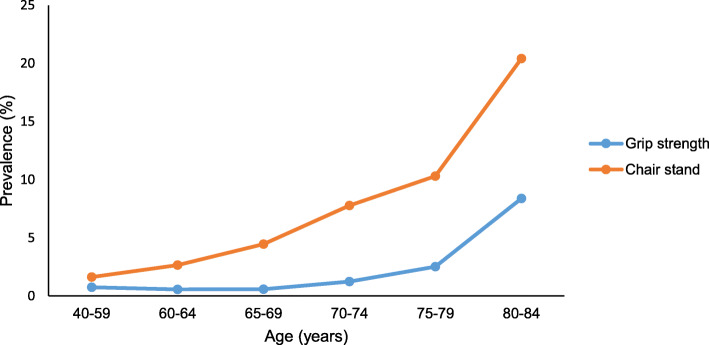
Prevalence of probable and confirmed sarcopenia based on grip strength or chair stand tests across age groups. The Tromsø Study 2015–16

Table 2 shows that using grip strength as the primary criteria resulted in 1.1% being classified as having probable sarcopenia among 40–84 year olds, while the corresponding prevalence for using chair stands was 3.9%. Total sample prevalence of confirmed sarcopenia only was 0.3% combined with ALM_height_^2^. Corresponding numbers for chair stands was 0.5% when combined with ALM_height_^2^. The total age-standardized sample prevalence for probable and confirmed sarcopenia combined was 1.3% using grip strength as the primary criteria and 4.4% using chair stand as the primary criteria. Grip strength-based prevalence of probable and confirmed sarcopenia remained fairly constant (0.6–0.7%) for ages 40–69, increasing to 1.2% in ages 70–74, to 2.5% in ages 75–79 and to 8.4% in ages 80+ years (Fig. 1). Prevalence of probable and confirmed sarcopenia defined by chair stands increased progressively from 1.6 to 2.7, 4.5, 7.8, 10.3 and 20.4% for ages 40–59, 60–64, 65–69, 70–74, 75–79 and 80+ years, respectively. Allowing any strength criteria to define sarcopenia resulted in a total sample prevalence of 4.5% (*n* = 205) with probable sarcopenia and 0.7% (*n* = 30) with confirmed sarcopenia (Table 2).

**Table 2:** EWGSOP2 sarcopenia prevalence based on grip strength, chair stand, or any criteria

EWGSOP2 algorithm	Sarcopenia classification	40–59 (*n* = 673)	60–64 (*n* = 717)	65–69 (*n* = 876)	70–74 (*n* = 643)	75–79 (*n* = 398)	80–84 (*n* = 191)	All (*n* = 3498)	Age-standardized prevalence (%)^a^
Grip strength + ALM_height_^2^	No sarcopenia	668 (99.26)	713 (99.44)	871 (99.43)	635 (98.76)	388 (97.49)	175 (91.62)	3450 (98.63)	–
	Probable sarcopenia	5 (0.74)	4 (0.56)	5 (0.57)	7 (1.09)	7 (1.76)	10 (5.24)	38 (1.09)	1.00
	Confirmed sarcopenia	0 (0.00)	0 (0.00)	0 (0.00)	1 (0.16)	3 (0.75)	6 (3.14)	10 (0.29)	0.26
Chair stands + ALM_height_^2^	No sarcopenia	662 (98.37)	698 (97.35)	837 (95.55)	593 (92.22)	357 (89.70)	152 (79.58)	3299 (94.31)	
	Probable sarcopenia	11 (1.63)	19 (2.65)	37 (4.22)	46 (7.15)	32 (8.04)	31 (16.23)	176 (5.03)	3.89
	Confirmed sarcopenia	0 (0.00)	0 (0.00)	2 (0.23)	4 (0.62)	9 (2.26)	8 (4.19)	23 (0.66)	0.51
Any strength criteria + ALM_height_^2^	No sarcopenia	659 (97.92)	695 (96.93)	833 (95.09)	586 (91.14)	350 (87.94)	140 (73.30)	3263 (93.28)	–
	Probable sarcopenia	14 (2.08)	22 (3.07)	41 (4.68)	53 (8.24)	36 (9.05)	39 (20.42)	205 (5.86)	4.54
	Confirmed sarcopenia	0 (0.00)	0 (0.00)	2 (0.23)	4 (0.62)	12 (3.02)	12 (6.28)	30 (0.86)	0.69

Numbers are n (%). *EWGSOP2* European Working Group on Sarcopenia in Older People revised definition, *ALM*_*height*_^*2*^ Appendicular Lean Mass relative to squared body height. ^a^Total sample prevalence standardized according to European Populations 2013. The Tromsø Study 2015–16

### Agreement between probable and confirmed sarcopenia status

**Fig. 2:**
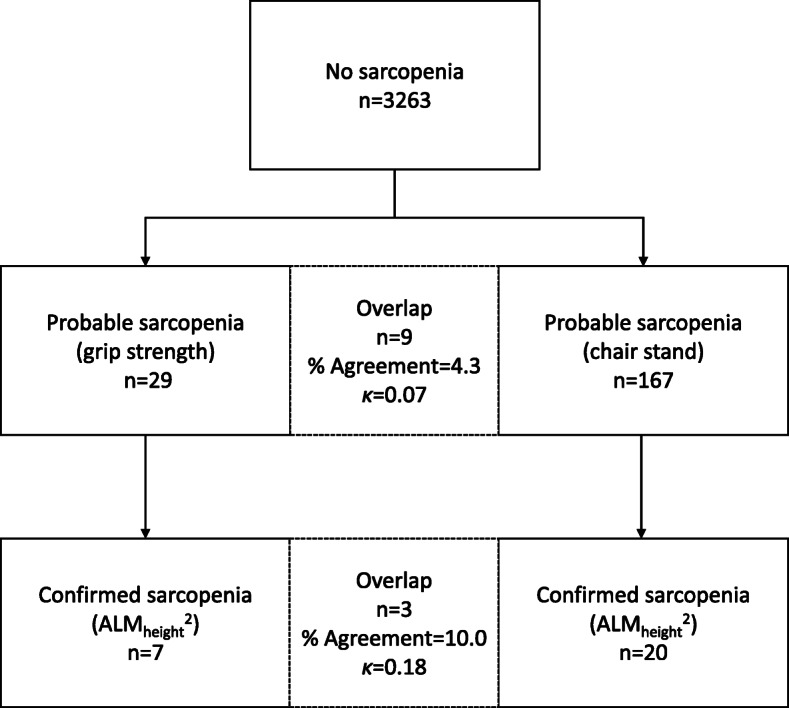
Agreement and overlap between cut-offs for probable and confirmed sarcopenia. κ = Cohen’s Kappa statistic, ALM_height_^2^ = Appendicular Lean Mass relative to squared body height. The Tromsø Study 2015–16

Figure 2 shows that 9 out of 205 (4.3%) participants overlapped between grip strength and chair stand criteria, indicating a very low level of agreement (*κ* = 0.07, 95% CI 0.02–0.12) between the two measures of probable sarcopenia. For subsequent determination of confirmed sarcopenia, 3 out of 30 (10.0%) participants overlapped, also indicating a very low level of agreement (*κ* = 0.18, 95% CI 0.00–0.36). Excluding overlapping participants, those with initial probable sarcopenia determined by grip strength were more likely to also have confirmed sarcopenia (24.1%) than those determined by chair stand (12.0%).

### Sarcopenia parameter cut-offs and sex differences

**Fig. 3:**
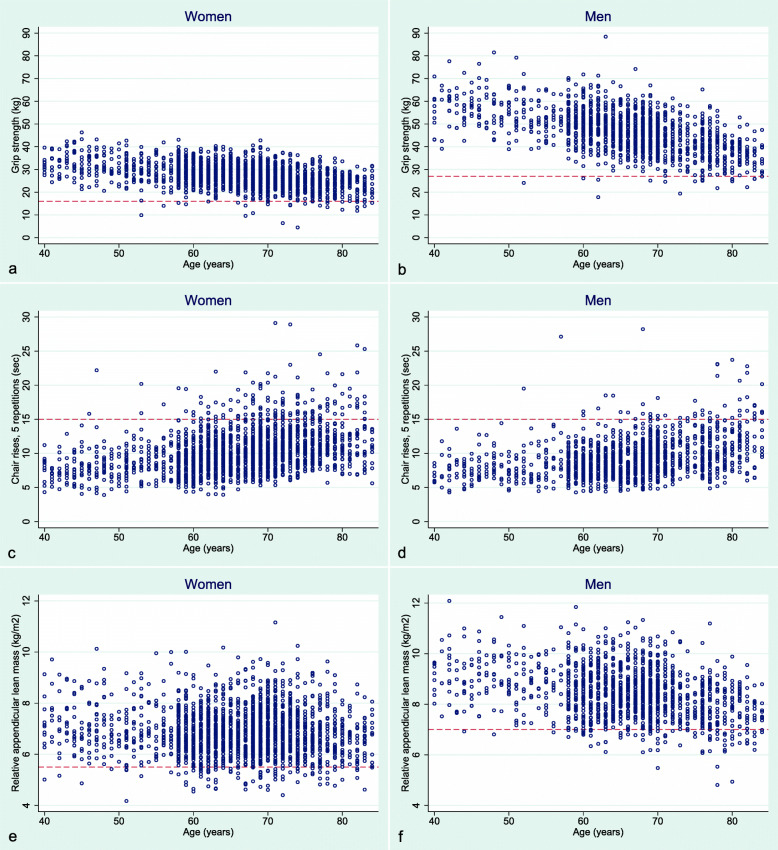
Presentation of study sample data for individual sarcopenic parameters stratified by sex across ages 40–84. **a** = grip strength among women with cut-off < 16 kg. **b** = grip strength among men with cut-off < 27 kg. **c** = five-repetition chair stands among women with cut-off > 15 s. **d** = five-repetition chair stands among men with cut-off > 15 s. **e** = appendicular lean mass relative to body height among women relative with cut-off < 5.5 kg/m^2^. **f** = appendicular lean mass relative to body height among men with cut-off < 7.0 kg/m^2^. Cut-offs are based on the EWGSOP2 definition and are represented by the red dashed line. The Tromsø Study 2015–16

Table 3 shows that there were no significant differences in proportions of men and women reaching the EWGSOP2 thresholds for grip strength (1.3 vs. 1.4%) or TUG (0.1 vs. 0.3%). The chair stand test defined a significantly larger proportion of women than men to have probable sarcopenia (7.2 vs. 3.6%, *p* < 0.001). Likewise, a larger proportion of women compared to men had slow walk speed (7.3 vs. 5.1%, *p* = 0.006). A higher proportion of men compared to women had low ALM_height_^2^ (5.9 vs. 4.4%, *p* = 0.038). Figure 3 further illustrates the sex differences in sarcopenia parameters, such as grip strength for women (Fig. 3a) and men (Fig. 3b), chair stands for women (Fig. 3c) and men (Fig. 3d), and ALM_height_^2^ for women (Fig. 3e) and men (Fig. 3f).

**Table 3:** Proportions of men and women crossing the sarcopenic threshold for each separate EWGSOP2 parameter

EWGSOP2 parameter	Men (*n* = 1451)	Women (*n* = 2047)	*P*
Grip strength	19 (1.31)	29 (1.42)	0.783
Chair stands	52 (3.58)	147 (7.18)	< 0.001
ALM_height_^2^	86 (5.93)	90 (4.40)	0.038
Walk speed^a, b^	74 (5.10)	150 (7.33)	0.006
TUG^a, c^	1 (0.07)	7 (0.34)	0.129

Numbers are n (%). *P*-values are age-adjusted by logistic regression. ^a^Walk speed and TUG had missing data for n = 2 and *n* = 3 participants respectively. ^b^EWGSOP2 thresholds for walkspeed is ≤ 0.8 m/s. ^c^ EWGSOP2 thresholds for TUG is ≥ 20 s. ALM_height_^2^ = Appendicular Lean Mass relative to squared body height, TUG = Timed-Up-and-GO test. The Tromsø Study 2015–16

### Probable sarcopenia measures as independent predictors of anthropometrics and body composition

The fully adjusted regression models (model 2) presented in Table 4 shows that sarcopenic participants based on grip strength weighed less (− 8.8 kg, 95% CI − 12.8 to − 4.7) and were shorter (− 6.1 cm, 95% CI − 8.0 to − 4.1) compared to non-sarcopenic participants. In contrast, sarcopenic participants based on chair stands weighed more (5.0 kg, 95% CI 3.0–6.9) than non-sarcopenic participants, while there was no height difference. Model 2 further shows that participants with probable sarcopenia according to grip strength had lower BMI (− 1.1 kg/m^2^, 95% CI − 2.5 to − 0.2), waist circumference (− 3.9 cm, 95% CI − 7.5 to − 0.3), and ALM_height_^2^ (− 0.5 kg/m^2^, 95% CI − 0.8 to − 0.2) compared to non-sarcopenic participants. In contrast, participants with probable sarcopenia according to chair stand performance had higher BMI (1.6 kg/m^2^, 95% CI 0.9 to 2.2), waist circumference (5.5 cm, 95% CI 3.8 to 7.3) and TBF% (2.7%, 95% CI 1.6 to 3.7) compared to their non-sarcopenic counterparts after adjusting for all covariates in model 2, including age and sex (Table 4).

**Table 4:** Independent associations for EWGSOP2 cut-offs for muscle strength with anthropometric data and body composition parameters

Independent variable	Dependent variable	Model 1		Model 2	
		Beta (95% CI)	*β*	Beta (95% CI)	*β*
Grip strength probable sarcopenia cut-off	Weight (kg)	−8.6 (−12.9 to − 4.4)	−0.067	−8.8 (− 12.8 to − 4.7)	−0.064
	Height (cm)	− 7.3 (− 10.0 to − 4.7)	−0.092	−6.1 (− 8.0 to − 4.1)	−0.071
	BMI (kg/m^2^)	−0.7 (− 1.9 to 0.6)	− 0.018	− 1.1 (− 2.5 to − 0.2)	− 0.028
	Waist circumference (cm)	−2.0 (− 5.5 to 1.6)	−0.018	−3.9 (− 7.5 to − 0.3)	−0.034
	Total body fat (%)	1.4 (−0.8 to 3.7)	0.021	0.1 (− 2.1 to 2.3)	0.002
	ALM_height_^2^ (kg/m^2^)	−0.7 (− 1.0 to − 0.3)	−0.065	− 0.5 (− 0.8 to − 0.2)	−0.046
Chair stand probable sarcopenia cut-off	Weight (kg)	2.1 (−0.1 to 4.2)	0.032	5.0 (3.0 to 6.9)	0.075
	Height (cm)	−2.9 (−4.2 to − 1.6)	−0.074	0.7 (−0.3 to 1.6)	0.017
	BMI (kg/m^2^)	1.7 (1.1 to 2.4)	0.093	1.6 (0.9 to 2.2)	0.082
	Waist circumference (cm)	5.3 (3.5 to 7.1)	0.098	5.5 (3.8 to 7.3)	0.100
	Total body fat (%)	5.2 (4.1 to 6.3)	0.154	2.7 (1.6 to 3.7)	0.100
	ALM_height_^2^ (kg/m^2^)	−0.3 (−0.4 to −0.1)	−0.052	0.1 (−0.0 to 0.3)	0.021

Numbers are unstandardized beta-coefficients (Beta) with 95% CIs and standardized beta coefficients (*β*). Coefficients indicate the resulting change on anthropometric and body composition data of reaching the probable sarcopenia cut-off point for either grip strength or chair stand tests. Model 1 is unadjusted. Model 2 (final model) is adjusted for sex, age, current smoker, CVD, current diabetes and educational level. ALM_height_^2^ = Appendicular Lean Mass relative to squared body height. The Tromsø Study 2015–16

### Associations between sarcopenia parameters and probable sarcopenia cut-off points

**Fig. 4:**
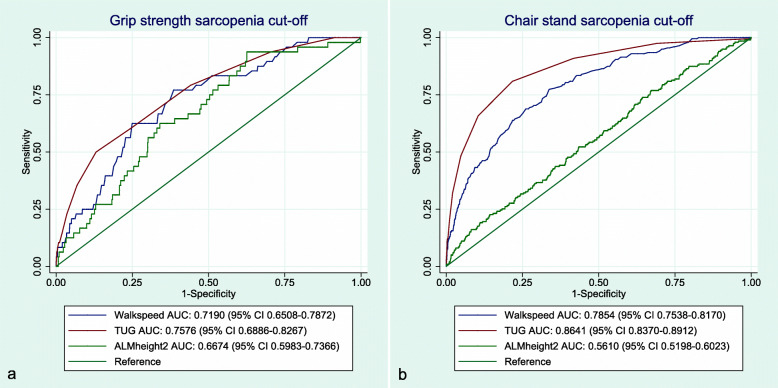
ROC-curves presenting sarcopenia parameter classification of grip strength and chair stand cut-offs for probable sarcopenia. a = classification of grip strength-based probable sarcopenia by walkspeed, TUG and ALM_height_^2^. b = classification of chair stand-based probable sarcopenia by walkspeed, TUG and ALM_height_^2^. AUC = Area Under the Curve, ALMheight2 = Appendicular Lean Mass relative to squared body height, TUG = Timed-Up-and-GO test. The Tromsø Study 2015-2016

The ROC-analysis showed no significant differences between the AUCs for walk speed, TUG or ALM_height_^2^ in relation to grip strength-based probable sarcopenia (Fig. 4a). For chair stand-based sarcopenia, the AUC for walk speed (0.79, 95% CI 0.75–0.82) was significantly larger than the AUC for ALM_height_^2^ (0.56, 95% CI 0.52–0.60), while the AUC for TUG was significantly larger (0.86, 95% CI 0.84–0.89) than all other sarcopenic parameters (Fig. 4b). In addition, the AUC for TUG was significantly larger when using chair stands (Fig. 4b) to classify probable sarcopenia compared to using grip strength (Fig. 4a; 0.76, 95% CI 0.69–0.83).

## Discussion

In this population-based study of 3498 women and men aged 40–84 years, the age-standardized prevalence of probable and confirmed sarcopenia was 1.3% when using grip strength, and 4.4% when using chair stands as primary sarcopenia defining measure. The chair stand test defined significantly more women than men as sarcopenic, and total sample prevalence based on chair stands was more than doubled across all age groups compared to using grip strength. Prevalence of chair stand-based probable sarcopenia also increased progressively from age 40–59, whereas the prevalence of grip strength-based probable sarcopenia increased notably first at age 70–74. In addition, we present novel data showing major differences in anthropometrics, body composition and physical function between participants depending on whether grip strength or chair stands were used to define probable sarcopenia.

Recently the UK Biobank reported a 5.3% prevalence of probable sarcopenia for 40–70 year olds [21], which differs from the 1.3% prevalence reported in the current study even though our participants were older. This could partly be influenced by instrumental differences, as we used the electronic version of the Jamar dynamometer compared to the hydraulic version commonly used by others. Grip strength differences between European regions might also influence this finding, as a similarly low prevalence (1.8%) of probable sarcopenia was recently found among older adults aged 70 years from northern Sweden [22]. Additionally, previous Tromsø Study findings indicate a temporal increase in muscle strength in the current study population [23].

Few large, population-based studies have compared the outcome of using grip strength or chair stand tests to define individuals as having probable sarcopenia according to the updated EWGSOP2 criteria. The Korean Frailty and Aging Cohort Study reported that among 2099 participants aged 70–84, 13.7% had grip strength-based probable sarcopenia and 13.6% had chair stand-based probable sarcopenia [24]. Their higher prevalence estimates are likely explained by the larger proportions of older participants in their study. Similar to our study, they found that significantly more women than men were defined as sarcopenic by chair stands, but conversely also found that significantly more men than women were sarcopenic by grip strength. We speculate that these differences could be partly explained by their higher inclusion of rural participants in contrast to the Tromsø Study which mainly involves urban residents. However, this sex discrepancy warrants elucidation, and it is also unclear why the total sample prevalence of probable sarcopenia was not higher in their study when using chair stands compared to grip strength, a finding now reported by us and by others previously [25].

After adjusting for multiple covariates, we found that participants with grip strength-based probable sarcopenia were shorter, lighter and less muscular than their non-sarcopenic counterparts, while participants with chair stand-based probable sarcopenia were generally heavier and more obese. This indicates that the two primary tests for sarcopenia may include individuals with contrasting features relative to non-sarcopenic individuals, even when accounting for major influencing factors such as age and sex. Compared to grip strength, chair stand performance likely depends more on the participant having sufficient leg muscle strength to lift their bodyweight [26], which may explain why the chair stand-based probable sarcopenia group were heavier compared the others. Adiposity may thus be a confounding factor in chair stand performance not necessarily associated with the frail and thin phenotype that is more common in participants with low grip strength. Longitudinal studies are required to evaluate whether the differences in these phenotypes have an impact on health outcomes. We also found that the EWGSOP2 cut-offs for chair stands defined significantly more women than men as sarcopenic, whereas for grip strength they did not. Unlike the grip strength cut-offs, the EWGSOP2 chair stand cut-offs do not differ between men and women [11]. Others have reported that women generally perform poorer than men in the chair stand test, potentially warranting sex-specific thresholds, although the differences between men and women are less distinct than for grip strength cut-off points [27].

Defining probable sarcopenia by either grip strength or chair stands resulted in different associations to the other sarcopenia parameters, where ALM_height_^2^ was more strongly associated with grip strength-based probable sarcopenia and TUG score was more strongly associated with chair stand-based probable sarcopenia. The relationship between chair stand and TUG performance is plausible given the assessment procedure similarities. The stronger association between grip strength and ALM_height_^2^ compared to chair stands and ALM_height_^2^ was somewhat surprising, given the chair stand test’s high correlation with leg muscle power, which in turn correlates with leg muscle mass [28, 29]. Although, that chair stand performance also relies on sensorimotor and psychological capabilities rather than just muscle strength may partly explain this finding [30]. The discrepancy between upper- and lower body strength measures, muscle mass and physical function could potentially produce selection bias in the EWGSOP2 algorithm steps when determining subsequent sarcopenia confirmation and severity in individuals. This is partly confirmed by the poor agreement and small overlap between grip strength and chair stand cut-offs, and that a larger proportion of participants with grip strength-based probable sarcopenia also had low ALM_height_^2^ and met the criteria for confirmed sarcopenia, compared to participants with chair stand-based probable sarcopenia. The lack of agreement between the assessment methods highlights the need to inform clinicians about the importance of using both grip strength and chair stand tests in sarcopenia screening, to properly identify all patients with probable sarcopenia according to the current EWGSOP2 definition.

The EWGSOP2 definitions specify that muscle strength can be determined by the grip strength test or the five-repetition chair stand test, suggesting interchangeability of these measures [11]. However, previous research have shown poor associations between upper- and lower body strength [13, 14], and we report differences in anthropometric parameters and physical function when using either the grip strength- or chair stand test to define probable sarcopenia. While usually preferred out of feasibility, studies show that grip strength testing may be insufficient in detecting associations between muscle strength, hospitalization and mortality [31]. Likewise, grip strength testing can be inadequate when evaluating the efficacy of intervention programs, while measures of leg strength are reportedly more sensitive to change following exercise regimens [6, 32]. However, a recent meta-analysis reported 31% reduced risk for all-cause mortality using grip strength compared to 14% reduced risk when using knee extension strength, adding further controversy as to whether upper-body or lower-body strength is more important for health outcomes [33].

The present study has some limitations that need addressing. Unfortunately, we did not have access to harder sarcopenia endpoints such as fractures or mortality. It would have been valuable to investigate how the differences between upper- and lower body strength testing would influence such outcomes. We did not perform in-depth analyses of the confirmed or severe sarcopenia stages due to relatively low numbers of participants in these groups. It would have been of value to explore whether probable sarcopenia classification based on grip strength or chair stands also influenced participant characteristics in later sarcopenia stages further down the EWGSOP2 algorithm. However, the EWGSOP2 state that probable sarcopenia often is enough to initiate cause assessment and interventions in clinical practice [11]. Additionally, we could only include 3498 out of 8346 participants because they had complete strength, physical function and body composition assessments. Although, we post-hoc analyzed 7745 participants with only grip strength and chair stand tests performed, and prevalence of probable sarcopenia, smoking, CVD, as well as mean BMI were very similar (see Additional file 2) to the current study results.

## Conclusions

The present study showed that age-standardized prevalence of probable and confirmed sarcopenia was relatively low (1.3–4.4%) in a population-based sample of community-dwelling Norwegians aged 40–84 years. The prevalence was more than twice as high across all ages when using chair stands compared to grip strength as the defining measure. Furthermore, the two strength measures defined individuals with contradictory anthropometrics, body composition and physical function to have probable sarcopenia. Researchers should further explore the consequences of using different strength measures in the EWGSOP2 algorithm, and evaluate whether these can be used interchangeably to define sarcopenia.

## Supplementary Information

**Additional file 1. **Participant Flow Chart. All inhabitants in Tromsø municipality aged 40 years and older (*N* = 32,591) were invited to the basic examination. A sub-sample (*N* = 13,028) was pre-marked for invitation to participate in the extended examination conducted approximately 2 weeks later. This sub-sample consisted of a) a randomized sample (*N* = 9925), and b) Tromsø 6 (2007–2008) participants who attended body composition, echocardiogram and eye examinations (*n* = 3103). A total of 21,083 women and men aged 40–99 years attended the basic examination (65%). A total of 8346 attended the extended examination (64% of the original pre-marked sub-sample and 90% of the sub-sample who also attended the basic examination). In the present study we included participants aged 40–84 years with complete data from physical function and body composition measurements (*n* = 3498).

**Additional file 2.** Comparison of study sample to larger sample with only physical function measures.

## Data Availability

The dataset supporting the article findings is available through application directed to the Tromsø Study by following the steps presented on their online page: https://en.uit.no/forskning/forskningsgrupper/sub?p_document_id=453582&sub_id=71247.

## Abbreviations

EWGSOP2: European Working Group on Sarcopenia in Older People (revised definition)
TUG: Timed-Up-and-GO
ALM: Appendicular Lean Mass
ALM_height_^2^: Appendicular Lean Mass (relative to squared body height)
ROC: Receiver Operating Characteristic
AUC: Area-Under-the-Curve
SPPB: Short Physical Performance Battery
4MWS: Four-Meter Walk Speed
DXA: Dual Energy X-ray Absorptiometry
TBF%: Total Body Fat Percentage
BMI: Body Mass Index
CVD: Cardiovascular Disease
