# Supplementary material for: Differences in sarcopenia prevalence between upper-body and lower-body based EWGSOP2 muscle strength criteria: the Tromsø study 2015–2016
Source: BMC Geriatr. 2020 Nov 10;20:461. doi: 10.1186/s12877-020-01860-w (PMC7654146; doi:10.1186/s12877-020-01860-w)
Supplement: Supplementary file 1 — Additional file 1. Participant Flow Chart. All inhabitants in Tromsø municipality aged 40 years and older (N = 32,591) were invited to the basic examination. A sub-sample (N = 13,028) was pre-marked for invitation to participate in the extended examination conducted approximately 2 weeks later. This sub-sample consisted of a) a randomized sample (N = 9925), and b) Tromsø 6 (2007–2008) participants who attended body composition, echocardiogram and eye examinations (n = 3103). A total of 21,083 women and men aged 40–99 years attended the basic examination (65%). A total of 8346 attended the extended examination (64% of the original pre-marked sub-sample and 90% of the sub-sample who also attended the basic examination). In the present study we included participants aged 40–84 years with complete data from physical function and body composition measurements (n = 3498). [file 12877_2020_1860_MOESM1_ESM.pdf]

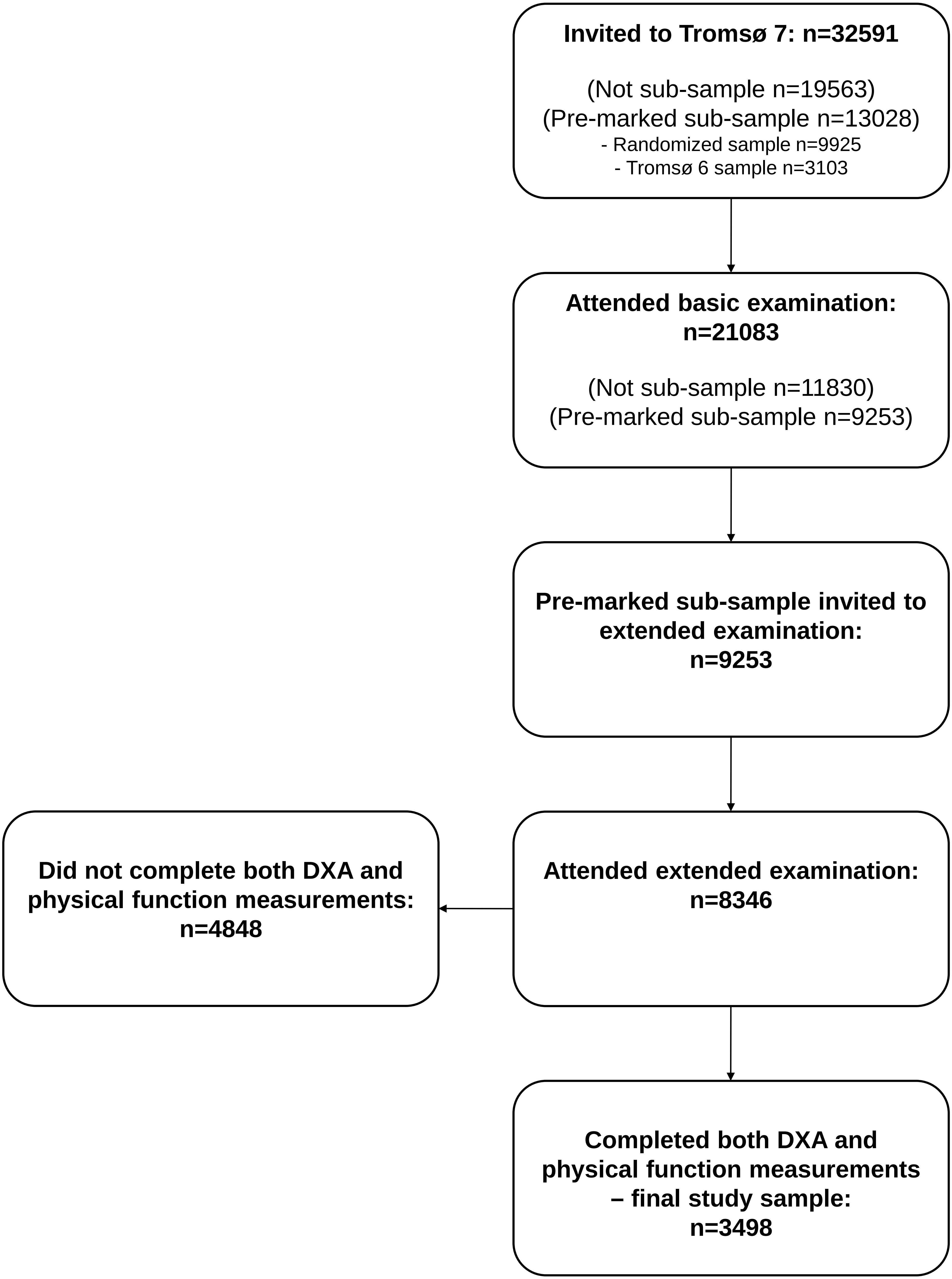
Invited to Tromsø 7: n=32591
(Not sub-sample n=19563)
(Pre-marked sub-sample n=13028)
- Randomized sample n=9925
- Tromsø 6 sample n=3103
Attended basic examination: n=21083
(Not sub-sample n=11830)
(Pre-marked sub-sample n=9253)
Pre-marked sub-sample invited to extended examination: n=9253
Attended extended examination: n=8346
Did not complete both DXA and physical function measurements: n=4848
Completed both DXA and physical function measurements – final study sample: n=3498
