# Supplementary material for: Differences in sarcopenia prevalence between upper-body and lower-body based EWGSOP2 muscle strength criteria: the Tromsø study 2015–2016
Source: BMC Geriatr. 2020 Nov 10;20:461. doi: 10.1186/s12877-020-01860-w (PMC7654146; doi:10.1186/s12877-020-01860-w)
Supplement: Supplementary file 2 — Additional file 2. Comparison of study sample to larger sample with only physical function measures. [file 12877_2020_1860_MOESM2_ESM.docx]

File name: Additional file 2

File format: Word table

Title of data: Comparison of study sample to larger sample with only physical function measures

Description of data:

| **Parameter** | **N = 7745** | **N = 3498** | **% Missing** |
| --- | --- | --- | --- |
| Grip strength-based probable sarcopenia (n, %) | 97 (1.3) | 48 (1.4) | - |
| Chair stand-based probable sarcopenia (n, %) | 384 (5.0) | 199 (5.7) | - |
| Current smoker (n, %) | 956 (12.5) | 388 (11.2) | 1.0 |
| Previous CVD (n, %) | 600 (8.0) | 265 (7.8) | 2.7 |
| BMI (kg/m^2^) | 27.3 (95% CI 27.2-27.4) | 27.1 (95% CI 27.0-27.3) | - |

CVD = Cardiovascular Disease (includes myocardial infarction and stroke)
